# Sex and External Size Specific Limitations in Assessing Bone Health From Adult Hand Radiographs

**DOI:** 10.1002/jbm4.10653

**Published:** 2022-06-29

**Authors:** Erin M.R. Bigelow, Robert W. Goulet, Antonio Ciarelli, Stephen H. Schlecht, David H. Kohn, Todd L. Bredbenner, Sioban D. Harlow, Carrie A. Karvonen‐Gutierrez, Karl J. Jepsen

**Affiliations:** ^1^ Department of Orthopaedic Surgery, Michigan Medicine University of Michigan Ann Arbor MI USA; ^2^ Department of Orthopaedic Surgery Indiana University School of Medicine Indianapolis IN USA; ^3^ Department of Biomedical Engineering University of Michigan Ann Arbor MI USA; ^4^ Biological and Materials Sciences, School of Dentistry University of Michigan Ann Arbor MI USA; ^5^ Department of Mechanical and Aerospace Engineering University of Colorado Colorado Springs Colorado Springs CO USA; ^6^ Department of Epidemiology, School of Public Health University of Michigan Ann Arbor MI USA

**Keywords:** biomechanics, hand radiograph, metacarpal, sex differences, strength

## Abstract

Morphological parameters measured for the second metacarpal from hand radiographs are used clinically for assessing bone health during growth and aging. Understanding how these morphological parameters relate to metacarpal strength and strength at other anatomical sites is critical for providing informed decision‐making regarding treatment strategies and effectiveness. The goals of this study were to evaluate the extent to which 11 morphological parameters, nine of which were measured from hand radiographs, relate to experimentally measured whole‐bone strength assessed at multiple anatomical sites and to test whether these associations differed between men and women. Bone morphology and strength were assessed for the second and third metacarpals, radial diaphysis, femoral diaphysis, and proximal femur for 28 white male donors (18–89 years old) and 35 white female donors (36–89+ years old). The only morphological parameter to show a significant correlation with strength without a sex‐specific effect was cortical area. Dimensionless morphological parameters derived from hand radiographs correlated significantly with strength for females, but few did for males. Males and females showed a significant association between the circularity of the metacarpal cross‐section and the outer width measured in the mediolateral direction. This cross‐sectional shape variation contributed to systematic bias in estimating strength using cortical area and assuming a circular cross‐section. This was confirmed by the observation that use of elliptical formulas reduced the systematic bias associated with using circular approximations for morphology. Thus, cortical area was the best predictor of strength without a sex‐specific difference in the correlation but was not without limitations owing to out‐of‐plane shape variations. The dependence of cross‐sectional shape on the outer bone width measured from a hand radiograph may provide a way to further improve bone health assessments and informed decision making for optimizing strength‐building and fracture‐prevention treatment strategies. © 2022 The Authors. *JBMR Plus* published by Wiley Periodicals LLC on behalf of American Society for Bone and Mineral Research.

## Introduction

Hand radiographs are used clinically for assessing bone health during growth^(^ ^1^, ^2^, ^3^, ^4^, ^5^, ^6^ ^)^ and aging,^(^ ^1^, ^7^, ^8^ ^)^ and for monitoring osteopenia, osteoporosis, and fracture risk at central anatomical sites like the proximal femur.^(^ ^9^, ^10^, ^11^, ^12^ ^)^ Many morphological parameters of the second metacarpal diaphysis have been derived as indices of bone health.^(^ ^1^, ^7^, ^8^ ^)^ However, these parameters have not been directly compared to experimentally measured strength of the metacarpal or other anatomical sites. Understanding the limitations of morphological parameters for estimating strength may have important clinical value for informed decision making. Further, as dimensionless morphological parameters are quantified in circumstances when calibrated hand radiographs are unavailable,^(^ ^7^ ^)^ it is also important to understand the limitations of using dimensionless parameters for estimating strength. The objectives of this study were to evaluate the extent to which various morphological parameters measured from hand radiographs relate to whole bone strength assessed at multiple anatomical sites and to test whether these associations differ between men and women.

## Materials and Methods

### Samples

Unfixed cadaveric second metacarpals, third metacarpals, radii, and femurs were collected from 28 white male donors (aged 18–89 years old) and 35 white female donors (aged 36–89+ years old) through the University of Michigan Anatomical Donations program (Ann Arbor, MI, USA), Science Care (Phoenix, AZ, USA), and Anatomy Gifts Registry (Hanover, MD, USA). With these sample sizes, we expected to detect correlation coefficients >0.5 with a power of 0.8 and significance level of 0.05,^(^ ^13^ ^)^ which is more than adequate for this study. Following procurement, all bones were wrapped in gauze soaked with phosphate buffered saline solution and stored frozen at −40°C. Human tissue use and handling were approved by the University of Michigan Institutional Biosafety Committee and declared exempt by the Institutional Review Board. Donors had no known conditions associated with disordered bone pathology. Handedness was unknown, and whereas it may lead to small morphology differences between dominant and nondominant hands,^(^ ^14^ ^)^ it would not impact the underlying structure–function relationship when studied across a large number of samples. Whole‐bone strength data were reported previously for the radii and femurs,^(^ ^15^, ^16^ ^)^ but are used herein to test for novel associations.

### Metacarpal morphology

**Fig. 1:**
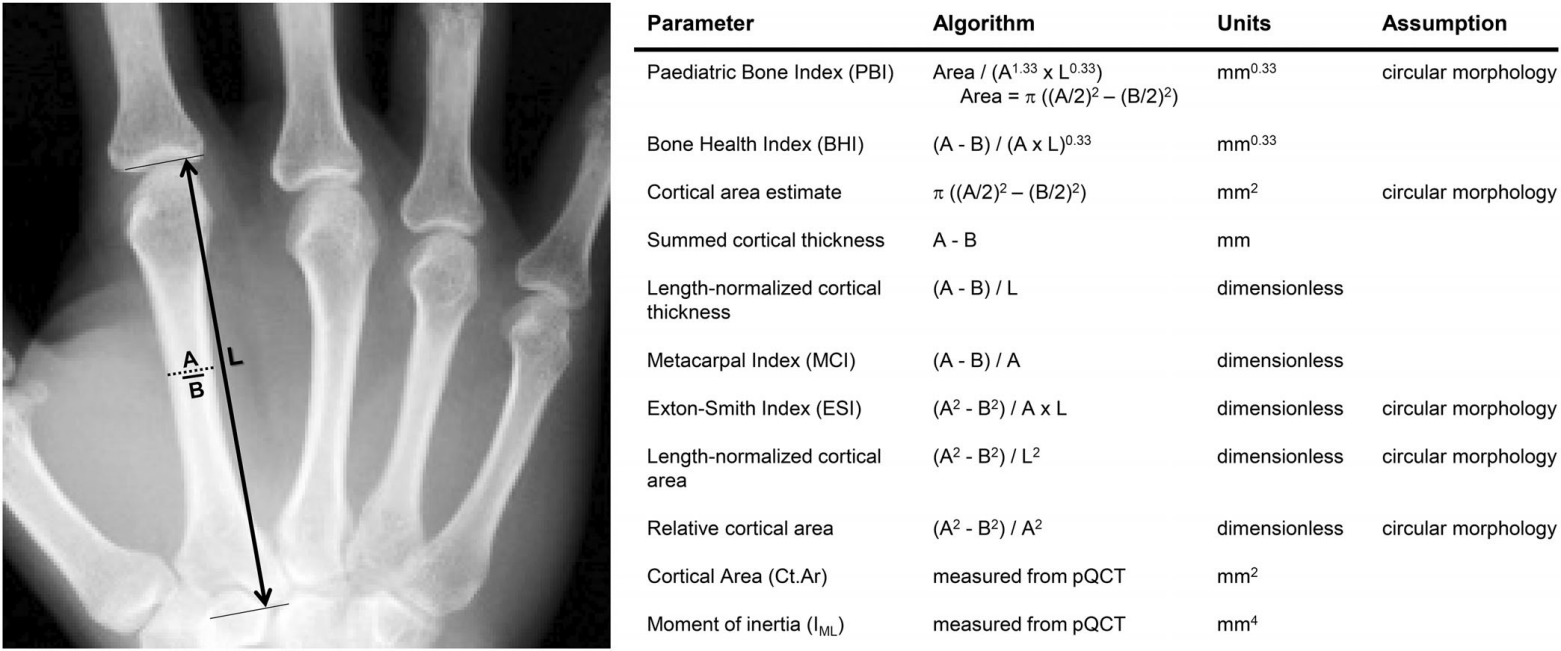
Illustration showing how outer (*A*) and inner (*B*) widths and length (L) were measured for the second metacarpal from hand radiographs. Nine morphological parameters derived from the hand radiographs along with the algorithm, units, and assumptions of circularity are indicated. Two morphological parameters (Ct.Ar, I_ML_) were measured directly from pQCT images. Ct.Ar = cortical area; I_ML_ = moment of inertia; pQCT = peripheral quantitative computed tomography.

Hand radiographs of intact left forearms were obtained using a portable X‐ray system with spatial and density calibration markers located adjacent to the hand. The X‐ray film was digitized at 1200 dots per inch (dpi) using a flatbed scanner (Epson Expression 10000XL; Seiko Epson Corp., Shiojiri‐Shi, Nagano‐Ken, Japan). In‐plane standards were used to calibrate measurements. This study focused on the second metacarpal because of its prevalent use in experimental and clinical bone health assessments.^(^ ^7^, ^11^, ^12^ ^)^ The length (L) of the second metacarpal was measured from the distal metaphysis to the most proximal portion of the proximal condyles (Fig. 1). Outer (A) and inner (B) widths were measured at sites located 40%, 50%, and 60% along the length, and then averaged over the three locations. A repeatability study of five bones, with five replicates each, resulted in coefficients of variation of 0.3% for length, 0.8% for outer width, and 2.3% for inner width, indicating high reproducibility of our methods for assessing morphology from hand radiographs. This region corresponds to the location of the middle loading points of the four‐point bending tests (see *Mechanical Testing* section below). Image analyses were conducted using MATLAB (The MathWorks, Inc., Natick, MA, USA). Edges were determined using an adaptive degree Savistzky‐Golay smoothing and differentiation script.^(^ ^17^, ^18^ ^)^ A manual check followed (EMRB) and edges were adjusted if necessary. Nine morphological indices, several of which are often reported in the literature,^(^ ^1^, ^7^, ^8^ ^)^ were calculated for each metacarpal using the following algorithms:

- Pediatric Bone Index (PBI) = Area/(A^1.33^ × L^0.33^), where Area = π ((A/2)^2^ − (B/2)^2^)
- Bone Health Index (BHI) = (A − B)/(A × L)^0.33^
- Cortical area estimate = π ((A/2)^2^ − (B/2)^2^)
- Summed cortical thickness = A − B
- Length‐normalized cortical thickness = (A − B)/L
- Metacarpal Index (MCI) = (A − B)/A
- Exton‐Smith Index (ESI) = A^2^ − B^2^/(A × L)
- Length‐normalized cortical area = (A^2^ − B^2^)/L^2^
- Relative cortical area = (A^2^ − B^2^)/A^2^

For the calculation of PBI, ESI, cortical area estimate, length‐normalized cortical area, and relative cortical area, it is assumed that the metacarpal has a circular cross‐sectional shape with concentric alignment of outer and inner surfaces. Following X‐ray imaging, the second metacarpal was dissected from the hand and imaged using a peripheral quantitative computed (pQCT) system (XCT 2000L; Stratec Medizintechnik, Pforzheim, Germany) to assess the actual midshaft cross‐sectional morphology. Images were acquired at 161‐μm in‐plane pixel resolution. Daily calibration scans were conducted to ensure consistent image quality. Images were analyzed with Image J (Momentmacro J; www.hopkinsmedicine.org/fae/mmacro.htm) as described.^(^ ^16^ ^)^ Briefly, images were thresholded to segment bone pixels from background and then analyzed for total area (Tt.Ar), cortical area (Ct.Ar), marrow area (Ma.Ar), moment of inertia about the ML axis (I_ML_), and outer widths in the medial‐lateral (D_ML_) and anteroposterior (D_AP_) directions. Variation in Ct.Ar measurements for other long bones has previously been reported between 0.3% and 2.6%.^(^ ^19^ ^)^

### Mechanical testing

**Fig. 2:**
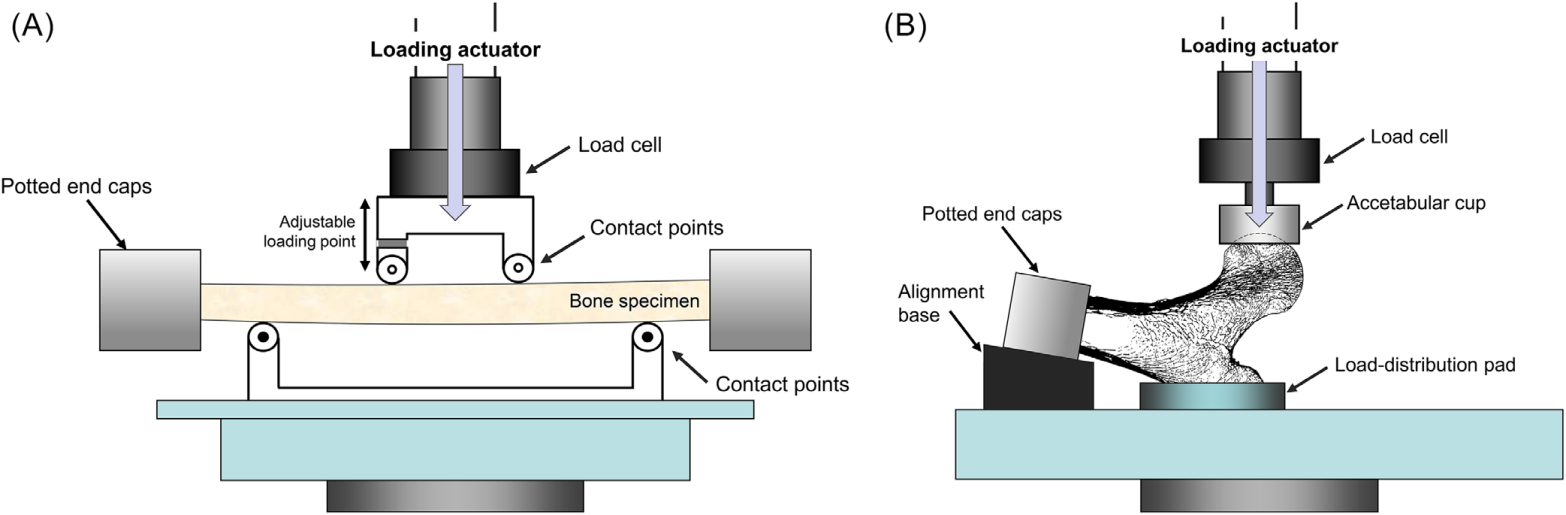
Schematic of the fixtures used to conduct (*A*) four‐point bending test of long bones and (*B*) fall‐to‐the side tests of proximal femurs. Note that the metacarpal tests were conducted without the potted end caps. The proximal femur shown is a sagittal section taken from a 3D high‐resolution image (nanocomputed tomography, 27 μm voxel size) and showing the internal cortical and trabecular architecture relative to the loading direction.

Left second and third metacarpals, radii, and femoral diaphyses were loaded to failure in four‐point bending (Fig. 2*A*) at a displacement rate of 0.1 mm/s using an Instron 8511 servo hydraulic materials testing system (Instron, Inc., Norwood, MA, USA). For all diaphyseal tests, the lower loading points were placed at 25% and 75% of the bone length. Upper loading points trisected the lower span. Bones underwent three pre‐yield loading trials before undergoing a failure test to ensure the bones were well seated on the test fixture. For the radius and femoral diaphysis, the distal and proximal metaphyseal regions were potted in square channels of acrylic resin (Ortho‐Jet BCA; Lang Dental, Wheeling, IL, USA) to prevent rotation during testing as described.^(^ ^15^ ^)^ The faces of the square channels aligned with the anterior, medial, posterior, and lateral faces of the bone. Metacarpals were loaded in the anterior‐to‐posterior direction; radii were loaded in the medial‐to‐lateral direction; and femurs were loaded in the posterior‐to‐anterior direction. These loading directions coincided with the natural curvature of the bones. Because bone length differed among samples, load–displacement curves were adjusted for test fixture geometry to calculate the maximum bending moment.^(^ ^19^, ^20^ ^)^ A validation test of the four‐point bending fixture was conducted and confirmed that the derived material modulus of aluminum cylinders was within 1% of textbook values.

The right proximal femurs were loaded to failure in the fall‐to‐the‐side configuration (Fig. 2*B*), as described.^(^ ^15^ ^)^ Briefly, the femur was sectioned at a location 16.5 cm from the superior point of the femoral head. The proximal femur shaft was internally rotated 15 degrees and potted in acrylic resin. Bones were held at a 10‐degree incline with respect to the horizontal surface using a custom‐made test fixture. Depression molds of the greater trochanter were made for each bone using a quick setting polyester putty (Bondo; 3M, Maplewood, MN, USA); these molds were used to distribute load to the greater trochanter during testing. Loading was applied through a metal acetabular cup that was sized to match each femoral head diameter. Each proximal femur was preloaded to 100 N through the acetabular cup before being loaded to failure at 100 mm/s. The maximum load prior to failure was determined from the load‐deflection graph. Validation tests confirmed that the mean displacement attributable to the test fixture design was 0.04 mm (0.02 mm–0.1 mm), which accounted for only 0.96% (0.56%–2.2%) of the total displacement of the fractured femurs.

Although multiple mechanical measures were calculated for each failure test described above, we focused exclusively on whole bone strength, which, for simplicity, is used generically in reference to the maximum bending moments calculated for the diaphyseal tests and the maximum load measured for the proximal femur tests. Due to technical issues, we did not have femoral diaphysis strength for three female donors and proximal femur strength for one female and one male donor.

### Statistical analysis

Four analyses were conducted using linear regression analysis and analysis of covariance (ANCOVA) to test for differences in the slopes and level (*y*‐intercepts) between the regressions constructed for the male and female cadavers (GraphPad Prism v.9.1.0; GraphPad Software, La Jolla, CA, USA). ANCOVA is a linear model that compares two (or more) groups while adjusting for one or more quantitative traits and is used by GraphPad Prism to compare two regression lines. First, metacarpal strength was regressed against each of the nine morphological measures derived from the hand radiograph to evaluate how well each parameter predicted strength and whether the associations varied by sex. Metacarpal strength was also regressed against cortical area (Ct.Ar) and moment of inertia (I_ML_) determined from pQCT data to test whether the associations improved with full knowledge of the cross‐sectional morphology. The regressions were also conducted after adjusting the morphological and strength data for age and centering on the average age of 65 years. Second, D_AP_, which is the outer width in the anteroposterior direction and out‐of‐plane relative to a hand radiograph, was regressed against D_ML,_ the outer width in the medial‐lateral direction, to further investigate the discrepancy observed for the morphology‐strength associations. Bland‐Altman analyses were conducted to test for systematic differences in estimating cross‐sectional morphology when assuming a circular versus an elliptical cross‐sectional shape.^(^ ^21^ ^)^ Third, second metacarpal strength was regressed against the strength measured for the third metacarpal, radial diaphysis, femoral diaphysis, and proximal femur. Finally, linear regression analysis was used to test how well metacarpal morphological parameters predicted strength measured at the third metacarpal, radial diaphysis, femoral diaphysis, and proximal femur. The regressions were also conducted after adjusting the morphological and strength data for age and centering on the average age of 65 years. Comparisons with *p* values <0.05 were considered significant.

## Results

**Fig. 3:**
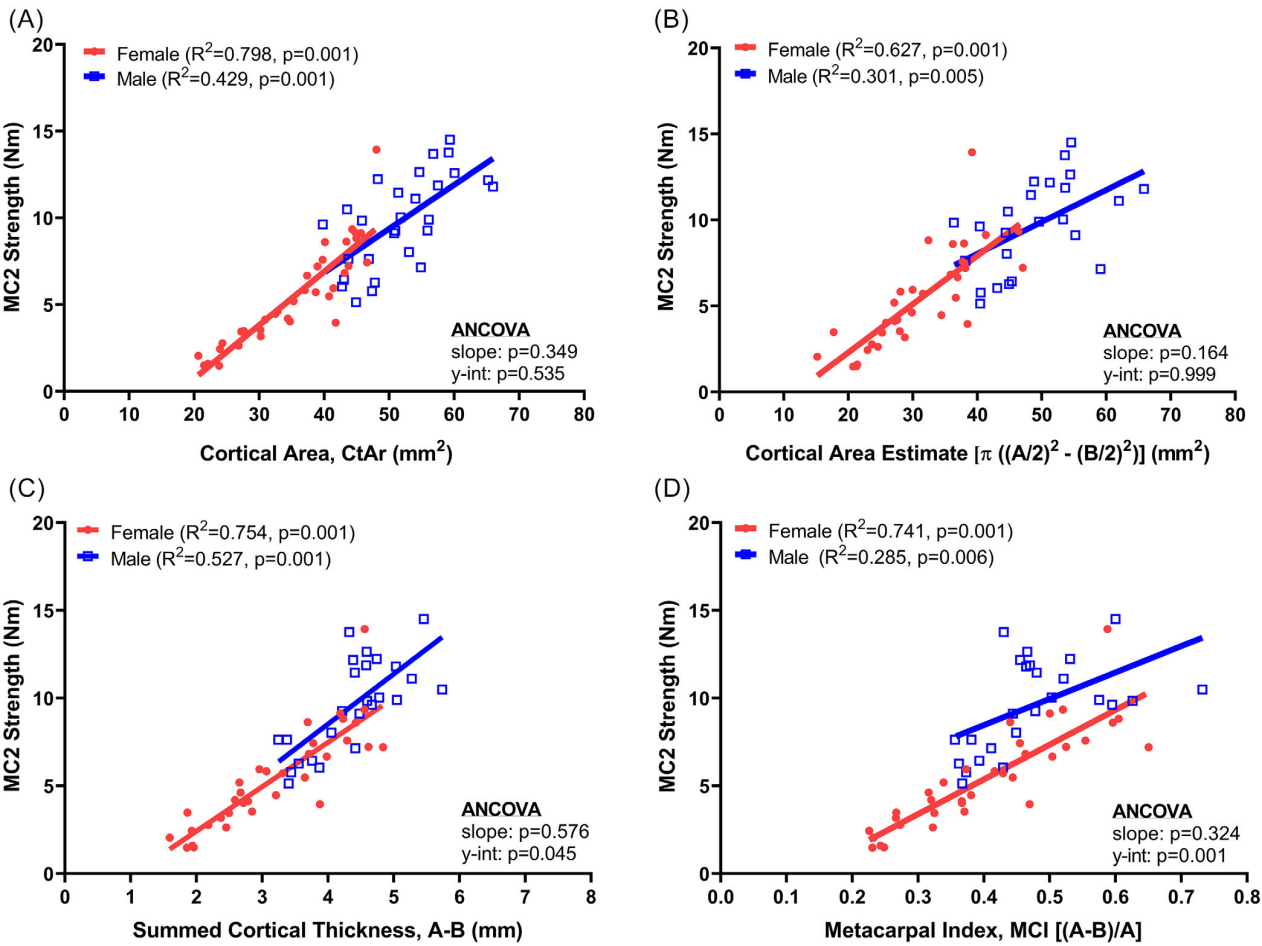
Linear regressions showing correlations between the strength of the MC2 and (*A*) cortical area measured from pQCT, (*B*) cortical area estimated from hand radiographs, (*C*) summed cortical thickness, and (*D*) MCI. MC2 = second metacarpal; MCI = metacarpal index; pQCT = peripheral quantitative computed tomography.

Summary data for all anthropometric, morphological, and mechanical properties are shown in Table S1. The strength of the second metacarpal was regressed against each of the nine morphological measures derived from the hand radiograph as well as cortical area (Ct.Ar) and moment of inertia (I_ML_) measured from pQCT (Table 1). The linear regressions indicated significant correlations for all parameters for females and for all but one, length‐normalized bone area, for males. For most parameters, the *R* ^2^ values for the regressions among females were nearly double those among males. Significant sex‐specific differences in the correlations were observed for all parameters except cortical area estimated from the hand radiographs and measured directly from pQCT. Notably, the summed cortical thickness (*A–B*) showed significant *R* ^2^ values for both males and females, and a small, albeit significant difference between sexes for the *y*‐intercept (*p* = 0.045, ANCOVA). Shown in Figure 3, the results of regressions for cortical area (Fig. 3*A,B*), summed cortical thickness (Fig. 3*C*), and MCI (Fig. 3*D*) illustrate how absolute values of cortical area but not relative measures of cortical area (eg, MCI) correlated with strength without a sex‐specific effect. The lowest *R* ^2^ values were for moment of inertia, indicating that this morphological measure explained the lowest amount of variation in metacarpal strength. Adjusting the data for age lowered the *R* ^2^ values for most regressions (Table S2) but did not meaningfully change the significance of the comparisons between regressions for males and females.

**Table 1:** Linear Regression Analysis Comparing Morphological Parameters and Whole Bone Strength for Female (*n* = 35) and Male (*n* = 28) Second Metacarpals

	Female		Male		ANCOVA	
Parameter	*R* ^2^	*p*	*R* ^2^	*p*	Slope	*y*‐intercept
Hand radiograph parameters						
Pediatric bone index, PBI	**0.732**	**0.001**	**0.504**	**0.001**	0.184	**0.007**
Bone health index, BHI	**0.746**	**0.001**	**0.422**	**0.001**	0.929	**0.001**
Cortical area estimate	**0.627**	**0.001**	**0.301**	**0.005**	0.164	0.999
Summed cortical thickness	**0.754**	**0.001**	**0.527**	**0.001**	0.576	**0.045**
Metacarpal index, MCI	**0.741**	**0.001**	**0.285**	**0.006**	0.324	**0.001**
Exton‐Smith index, ESI	**0.661**	**0.001**	**0.336**	**0.002**	0.802	**0.008**
Length normalized bone area	**0.425**	**0.001**	0.098	0.127	0.134	**0.004**
Relative bone area	**0.637**	**0.001**	**0.262**	**0.009**	0.990	**0.001**
Length normalized cortical thickness	**0.707**	**0.001**	**0.379**	**0.001**	0.999	**0.001**
pQCT parameters						
Cortical area, Ct.Ar	**0.798**	**0.001**	**0.429**	**0.001**	0.349	0.535
Moment of inertia, I_ML_	**0.407**	**0.001**	**0.148**	**0.044**	**0.001**	n/a

Bold values indicate significant correlations or differences in the slope and y‐intercept between male and female regressions (ANCOVA).

ANCOVA = analysis of covariance.

**Fig. 4:**
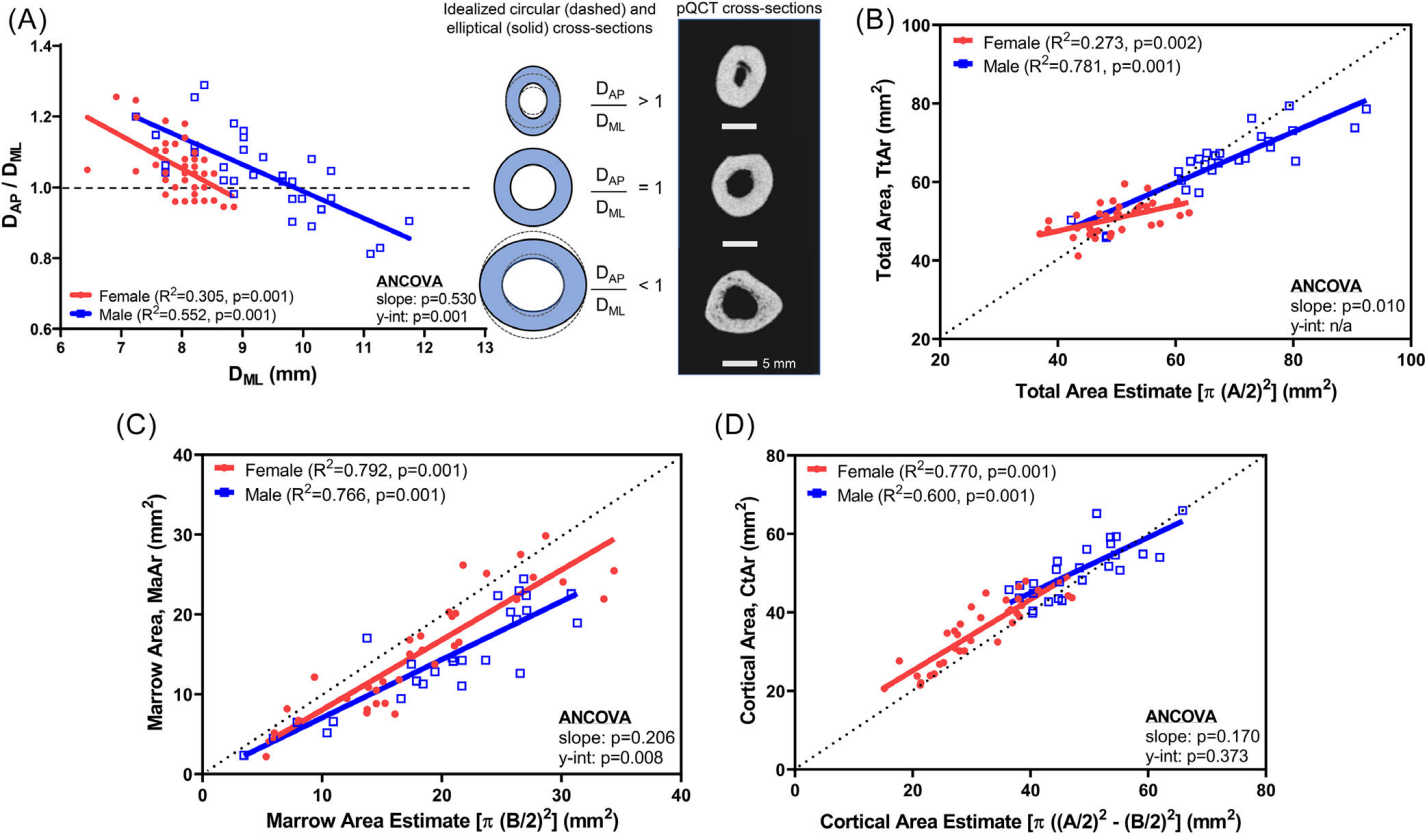
Linear regressions showing correlations between (*A*) D_AP_/D_ML_ and D_ML_ measured from pQCT of the second metacarpal, (*B*) Tt.Ar measured from pQCT and Tt.Ar estimated from hand radiographs, (*C*) Ma.Ar measured from pQCT and Ma.Ar estimated from hand radiographs, and (*D*) Ct.Ar measured from pQCT and Ct.Ar estimated from hand radiographs. Ct.Ar = cortical area; D_AP_ = outer width in the anteroposterior direction; D_AP_/D_ML_ = circularity ratio; D_ML_ = outer width in the medial‐lateral direction; Ma.Ar = marrow area; pQCT = peripheral quantitative computed tomography; Tt.At = total area.

We next examined how structural information derived from dimensions measured in the medial‐lateral (ML) plane related to structural information in the anterior‐posterior (AP) direction, which is out‐of‐plane and thus not measurable from a hand radiograph. First, the ratio, D_AP_/D_ML_ measured from pQCT cross‐sections was calculated as a measure of cross‐sectional shape, where D_AP_/D_ML_ = 1 indicates a circular cross‐section. On average, the metacarpals were slightly elliptical, with D_AP_/D_ML_ values of 1.05 ± 0.12 for males and 1.05 ± 0.09 for females (*p* = 0.543, *t* test). However, both males and females showed a significant negative association between D_AP_/D_ML_ and D_ML_ (Fig. 4*A*), indicating that the circularity of the metacarpal cross‐section depended on the outer width measured in the ML direction. For low values of D_ML_ (narrower bones), the metacarpal showed a D_AP_/D_ML_ ratio >1, indicating that narrower metacarpals were elliptical and “tall” relative to the width measured on a hand radiograph. At higher values of D_ML_ (wider bones), females showed a more circular cross‐section with the D_AP_/D_ML_ ratio close to 1, whereas males showed a D_AP_/D_ML_ ratio less than 1, indicating that wider male metacarpals were elliptical and “flatter.”

**Fig. 5:**
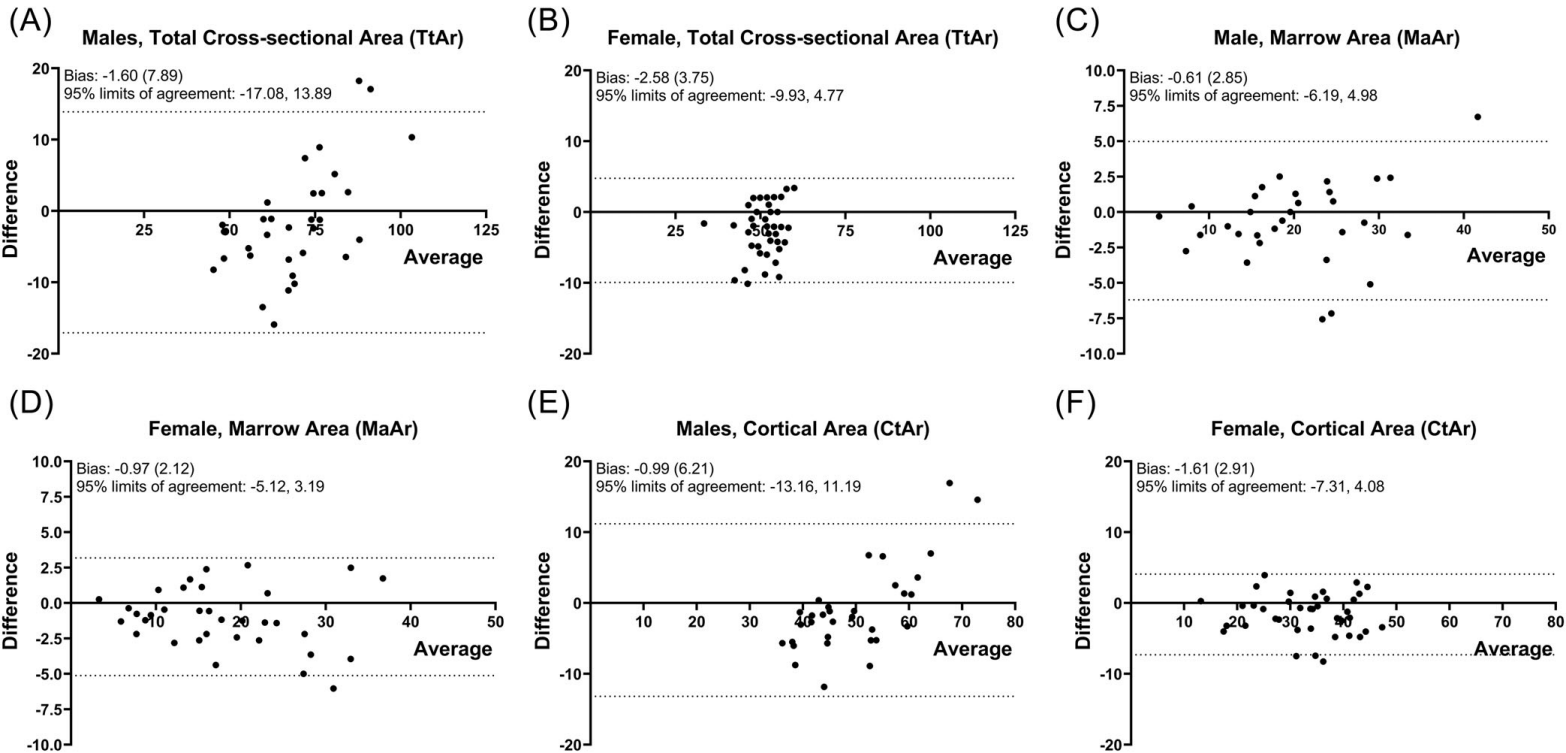
Bland‐Altman plots comparing circular versus elliptical formulas for (*A*,*B*) total cross‐section area, (*C*,*D*) marrow area, and (*E*,*F*) cortical area. Male and female data are shown for each morphological parameter.

**Fig. 6:**
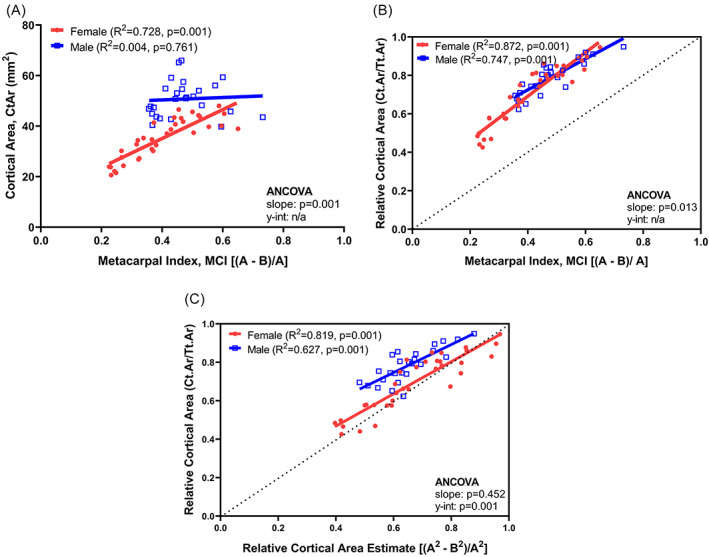
Linear regressions showing correlations between (*A*) cortical area measured from pQCT and metacarpal index measured from hand radiographs, (*B*) relative cortical area measured from pQCT and metacarpal index measured from hand radiographs, and (*C*) relative cortical area measured from pQCT and relative cortical area estimated from hand radiographs. pQCT = peripheral quantitative computed tomography.

The external size dependent shape differences resulted in discrepancies in the extent to which total area, cortical area, and marrow area estimated from hand radiographs by assuming a circular cross‐section correlated with actual measures of cross‐sectional morphology (Fig. 4*B–D*). For males and females, estimating cross‐sectional morphology from hand radiographs underestimated the actual Tt.Ar for narrower metacarpals and overestimated Tt.Ar and Ma.Ar for wider metacarpals. Surprisingly, estimates of cortical area using a circular approximation correlated well with the actual cortical area, with the regression falling closely along the identity line (*x* = *y*). Bland‐Altman plots (Fig. 5) were used to test for systematic differences in the agreement between estimating cross‐sectional morphology (Tt.Ar (Fig. 5*A,B*), Ma.Ar (Fig. 5*C,D*), Ct.Ar (Fig. 5*E,F*)) assuming a circular or elliptical cross‐sectional shape. For males, the two methods showed relatively small fixed‐bias for Tt.Ar (bias = −1.6; 2.3% of mean Tt.Ar), Ma.Ar (bias = −0.61; 3.1%, of mean Ma.Ar), and Ct.Ar (bias = −0.99; 2.0% of mean Ct.Ar), but large proportional bias for Tt.Ar and Ct.Ar, consistent with the dependence of cross‐sectional shape on outer width. In contrast, females showed larger fixed‐bias for Tt.Ar (bias = −2.58; 5.2% of mean Tt.Ar), Ma.Ar (bias = −0.97; 5.5% of mean Ma.Ar), and Ct.Ar (bias = −1.61; 5.0% of mean Ct.Ar), but did not show the proportional bias observed for males. Given this external size dependent bias, MCI correlated significantly with Ct.Ar for women but not men (Fig. 6*A*); MCI correlated significantly with relative cortical area from pQCT but with a substantial offset from the identity (*x* = *y*) line (Fig. 6*B*); and relative cortical area from hand radiographs correlated significantly with relative cortical area from pQCT but with a significant sex‐specific effect (Fig. 6*C*).

**Fig. 7:**
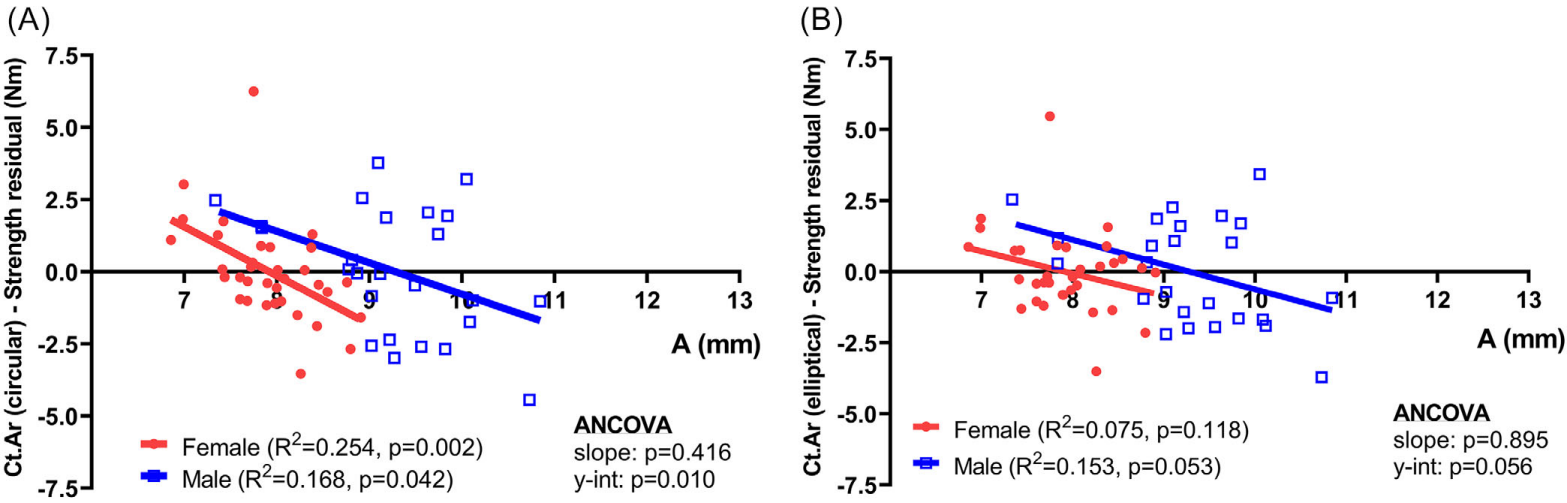
Correlations between the residuals from the (*A*) cortical area (circular) – strength regression and outer width, and (*B*) cortical area (elliptical) – strength regression and outer width.

Partial regression analysis was used to determine the extent to which dimensional information from hand radiographs predicted metacarpal strength (Fig. 7). Residuals from the regression between the cortical area estimate and strength showed significant negative correlations relative to A (outer bone width) for males and females (Fig. 7*A*), indicating that strength was overestimated for narrower bones but underestimated for wider bones using the cortical area estimate. We then tested whether using an elliptical formula for cortical area (π a b, where a = width along the ML axis, b = width along the AP axis) would improve the correlation between cortical area and strength and reduce the systematic bias. Empirical equations derived from the linear regressions between the shape factor (D_AP_/D_ML_, circularity) and the outer and inner widths measured from the pQCT images were used to estimate the out‐of‐plane (AP) widths for the outer and inner surfaces based on the outer (A) and inner (B) widths measured from the hand radiographs (Fig. 1). This enabled the use of elliptical formulas to estimate cross‐sectional area from hand radiographs. The correlation between the residuals from the cortical area‐strength regression and outer width (A) were no longer significant for females and modestly improved for males (Fig. 7*B*).

**Fig. 8:**
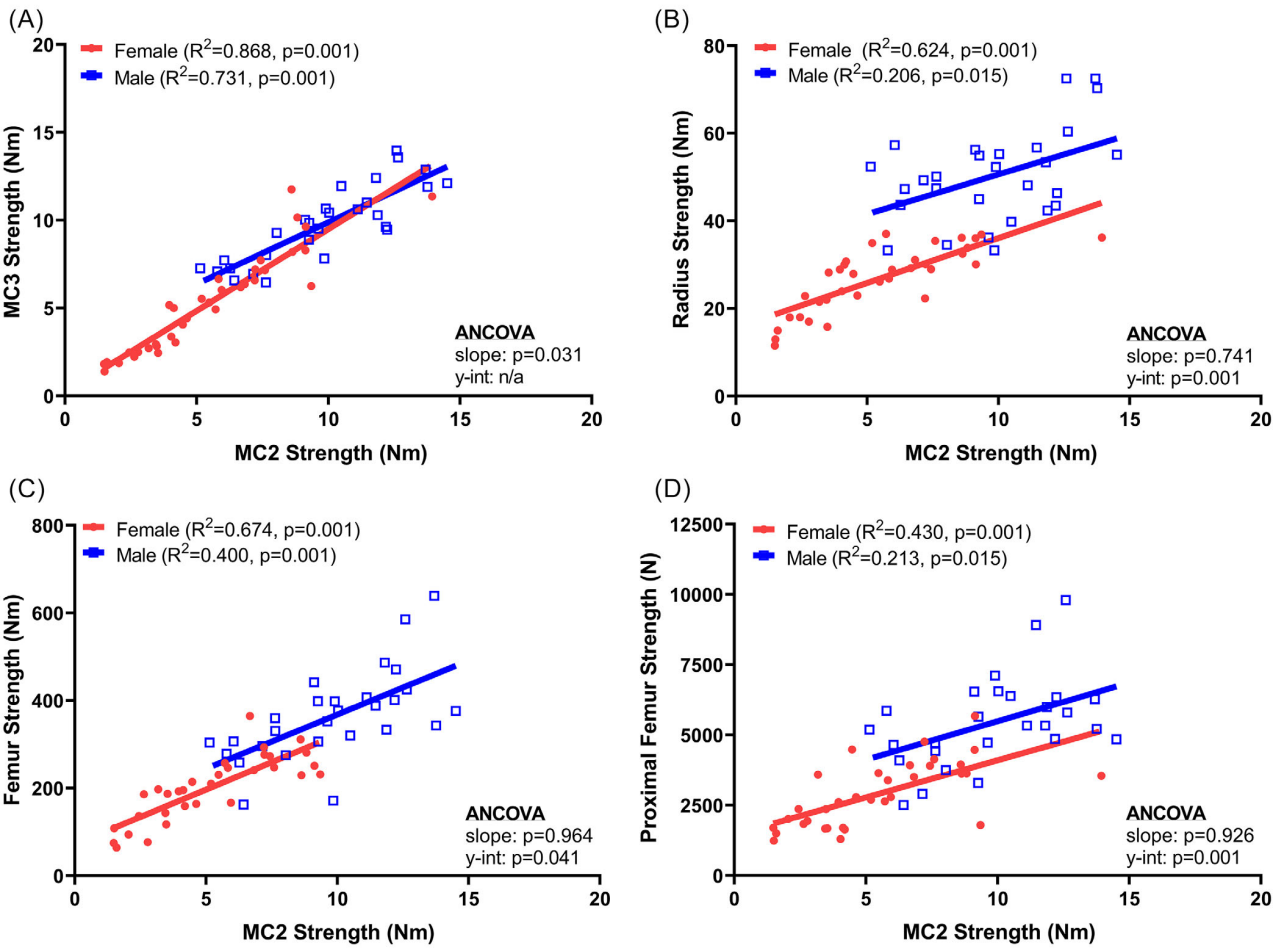
Linear regressions between MC2 strength and the strength measured for the (*A*) MC3, (*B*) radius, (*C*) femoral diaphysis, and (*D*) proximal femur. MC2 = second metacarpal; MC3 = third metacarpal.

The strength of the second metacarpal correlated significantly with the strength measured for the third metacarpal (Fig. 8*A*), radial diaphysis (Fig. 8*B*), femoral diaphysis (Fig. 8*C*), and proximal femur (Fig. 8*D*) for both males and females (Fig. 8). Significant sex differences in either the slopes or *y*‐intercepts were observed for each regression, indicating that associations between the second metacarpal strength and strength at other anatomical sites varied with sex. In general, males showed stronger femoral diaphyses, radial diaphyses, and proximal femurs relative to metacarpal strength compared to females. A small, but significant difference in the slope of the regression between the second and third metacarpal strengths was observed, but strength overlapped greatly between males and females suggesting the sex differences were minimal on a practical basis.

Last, we assessed the associations between morphological parameters derived from the second metacarpal with strength measured at the third metacarpal, radial diaphysis, femoral diaphysis, and proximal femur (Table 2*A,B*). Females showed significant correlations for all nine morphological parameters and two pQCT measures and strength of the third metacarpal diaphysis, radial diaphysis, femoral diaphysis, and proximal femur. In contrast, these morphological and pQCT measures for males correlated significantly with strength of the third metacarpal but only a few of the measures correlated significantly with strength of the radial diaphysis, femoral diaphysis, while none were correlated with the proximal femur. Adjusting for age lowered the *R* ^2^ values in most cases but did not meaningfully alter the overall outcome (Table S3).

**Table 2:** Linear Regression Analysis Comparing various Morphological Parameters to Whole Bone Strength Across Multiple Sites for (*A*) Female and (*B*) Male Cadaveric Bones

(*A*) Female	MC3		Radius		Femur		Proximal femur	
Parameter	*R* ^2^	*p*	*R* ^2^	*p*	*R* ^2^	*p*	*R* ^2^	*p*
Hand radiographs								
Pediatric bone index, PBI	**0.704**	**0.001**	**0.626**	**0.001**	**0.776**	**0.001**	**0.463**	**0.001**
Bone health index, BHI	**0.732**	**0.001**	**0.575**	**0.001**	**0.767**	**0.001**	**0.464**	**0.001**
Cortical area estimate	**0.538**	**0.001**	**0.548**	**0.001**	**0.611**	**0.001**	**0.476**	**0.001**
Summed cortical thickness	**0.724**	**0.001**	**0.580**	**0.001**	**0.748**	**0.001**	**0.485**	**0.001**
Metacarpal index, MCI	**0.763**	**0.001**	**0.550**	**0.001**	**0.757**	**0.001**	**0.435**	**0.001**
Exton‐Smith index, ESI	**0.614**	**0.001**	**0.594**	**0.001**	**0.743**	**0.001**	**0.430**	**0.001**
Length normalized bone area	**0.380**	**0.001**	**0.393**	**0.001**	**0.624**	**0.001**	**0.317**	**0.001**
Relative bone area	**0.723**	**0.001**	**0.463**	**0.001**	**0.761**	**0.001**	**0.371**	**0.001**
Length normalized cortical thickness	**0.676**	**0.001**	**0.566**	**0.001**	**0.760**	**0.001**	**0.449**	**0.001**
pQCT								
Cortical area, Ct.Ar	**0.754**	**0.001**	**0.686**	**0.001**	**0.640**	**0.001**	**0.461**	**0.001**
Moment of inertia, I_ML_	**0.263**	**0.002**	**0.445**	**0.001**	**0.187**	**0.013**	**0.206**	**0.007**

(*B*) Male	MC3		Radius		Femur		Proximal femur	
Parameter	*R* ^2^	*p*	*R* ^2^	*p*	*R* ^2^	*p*	*R* ^2^	*p*
Hand radiographs								
Pediatric bone index, PBI	**0.542**	**0.001**	0.009	0.656	**0.195**	**0.027**	0.125	0.090
Bone health index, BHI	**0.453**	**0.001**	0.002	0.824	0.097	0.130	0.139	0.073
Cortical area estimate	**0.364**	**0.001**	**0.222**	**0.017**	**0.325**	**0.003**	0.024	0.473
Summed cortical thickness	**0.577**	**0.001**	0.007	0.686	**0.180**	**0.035**	0.141	0.071
Metacarpal index, MCI	**0.285**	**0.006**	0.045	0.308	0.019	0.515	0.139	0.073
Exton‐Smith index, ESI	**0.402**	**0.001**	0.032	0.390	**0.214**	**0.020**	0.056	0.266
Length normalized bone area	**0.159**	**0.048**	0.104	0.116	**0.196**	**0.027**	0.001	0.893
Relative bone area	**0.298**	**0.005**	0.018	0.524	0.011	0.620	0.100	0.133
Length normalized cortical thickness	**0.432**	**0.001**	0.001	0.901	0.131	0.075	0.098	0.136
pQCT								
Cortical area, Ct.Ar	**0.363**	**0.001**	**0.184**	**0.023**	**0.271**	**0.005**	0.065	0.199
Moment of inertia, I_ML_	**0.218**	**0.012**	**0.394**	**0.001**	**0.286**	**0.003**	0.088	0.132

Bold values indicate significant correlations or differences in the slope and *y*‐intercept between male and female regressions (ANCOVA).

ANCOVA = analysis of covariance; MC3 = third metacarpal.

## Discussion

To the best of our knowledge, this is the first report of sex‐specific differences in associations between commonly used morphological parameters derived from hand radiographs and experimentally measured strength. Hand radiographs are used to evaluate bone health during growth^(^ ^1^, ^2^, ^3^, ^4^, ^5^ ^)^ and aging,^(^ ^7^, ^8^, ^9^, ^10^, ^11^, ^12^ ^)^ and to determine whether a condition or disease has affected bone strength.^(^ ^6^ ^)^ Thus, it is important to know how the parameters used to assess bone health relate to metacarpal strength and strength at other anatomical sites. Prior work reported morphological parameters as bone health indices but did not relate these parameters to experimentally measured strength. In this study, cortical area was shown to have strong associations with metacarpal strength for both sexes and without sex‐specific differences in the level (*y*‐intercept) or slope of the linear regressions (Table 1, Fig. 3), suggesting that a simple measure of the amount of bone at the mid‐shaft could be used as a consistent indicator of bone health. Other morphological parameters were not well correlated with strength in one or both sexes, or showed a significant sex‐specific effect. Although the age range differed slightly between male (18–89 years) and female (36 89+ years) donors, repeating the analyses using age‐adjusted data reduced some *R* ^2^ values between the morphological traits and mechanical properties but did not meaningfully affect the overall outcomes of the study (Tables S2 and S3).

Although the resistance of long bones to deformation under bending is generally thought to depend on cross‐sectional measures of bone tissue distribution like moment of inertia or section modulus following classical beam theory, this association only holds for structures that are long relative to outer width.^(^ ^20^, ^22^ ^)^ Metacarpals are short beams and stress under bending loads is largely dependent on shear stress. In short beams, shear stresses are associated with the amount of material present (eg, cortical area) and the ratio of loading span to beam cross‐sectional dimensions, rather than the distribution of that material,^(^ ^23^ ^)^ which may explain why moment of inertia showed weak associations with strength and why cortical area, whether measured directly from pQCT or estimated from plain film radiographs, showed strong associations without sex‐specific differences in the level or slope of the regressions. Thus, the sex‐independent association between Ct.Ar and strength is consistent with engineering studies and helps explain why male metacarpals are, on average, stronger than female metacarpals. Although male long bones tend to be wider than females, a morphological difference that is generally attributed to different periosteal expansion rates during puberty,^(^ ^5^ ^)^ male long bones also have greater cortical area than females even after adjusting for differences in body size and external bone size.^(^ ^24^ ^)^ Given the wide range in ages of our sample collection, the significant cortical area‐strength associations reported herein suggested that the greater metacarpal strength of males compared to females results from sex‐specific differences in the amount of bone mass accumulated by adulthood and/or maintained with aging. A similar argument can be made for the summed cortical thickness with the caveat that a small but significant difference in the *y*‐intercept was found between men and women. Thus, the current study helps explain how known differences in morphology of male and female metacarpals translate to strength differences.

A novel finding of this study was that cross‐sectional shape of the metacarpal varied with outer width measured along the ML axis. Although most studies assume that metacarpals have a circular shape, midshaft morphological parameters are better estimated using elliptical formulas.^(^ ^25^ ^)^ However, use of elliptical formulas requires knowledge of the out‐of‐plane shape of the metacarpal, which cannot be directly measured from hand radiographs. We found a significant correlation between the D_AP_/D_ML_ ratio and outer bone size in the ML direction (Fig. 4*A*), indicating that the out‐of‐plane shape may be estimated from dimensional information that is available on a hand radiograph. Our analyses indicated that narrower metacarpals tended to be taller whereas wider metacarpals tended to be flatter in the AP dimension. A similar out‐of‐plane phenomenon was reported for the femoral neck^(^ ^26^ ^)^; however, for this structure, narrower femoral necks tended to have a more circular cross‐section whereas wider femoral necks tended to be more elliptical.

Although Ct.Ar was a consistent predictor of strength, this morphological parameter was not without limitations. The external size dependent difference in metacarpal shape appeared to contribute to a systematic bias, underestimating cross‐sectional morphology and strength for narrower metacarpals and overestimating them for wider metacarpals when a circular cross‐sectional shape is assumed. The residuals from the Ct.Ar‐strength regression ranged from 0 to ~2.5 Nm, which is roughly 25% of the overall strength for our male and female cohorts, indicating that the error in estimating strength assuming a circular cross‐section was substantial. The association between cross‐sectional shape and ML width creates an opportunity to improve the use of hand radiographs for evaluating bone health by identifying correction factors that would enable the use of elliptical formulas for measuring morphology. The empirically based elliptical formulas for cortical area reduced the bias when estimating strength (Fig. 7*B*). Admittedly, the empirical adjustment for estimating cortical area using elliptical algorithms was not ideal since it involved two different imaging technologies (pQCT, hand radiographs). Nevertheless, the adjustment did reduce the systematic bias for estimating strength, particularly for females, providing evidence that out‐of‐plane shape differences may be contributing to the systematic bias. Given the dependence of circularity on outer width measured from a hand radiograph, our work suggests that additional studies could identify correction factors more rigorously and with the intention of improving bone strength assessments.

Metacarpal strength correlated significantly with strength at other anatomical sites, including the proximal femur which is a high‐risk fracture site. This outcome is consistent with prior work showing that metacarpal indices are associated with femoral neck fractures.^(^ ^10^, ^27^, ^28^, ^29^, ^30^, ^31^, ^32^, ^33^, ^34^, ^35^, ^36^, ^37^, ^38^, ^39^, ^40^, ^41^ ^)^ Our cadaveric cohort was established with a wide range of ages and body sizes to ensure large variation in metacarpal morphology and strength. The strength measures for the diaphyseal tests incorporated body size effects by adjusting the load and deformation data for test fixture geometry which varied with bone length. This adjustment could not be conducted for the proximal femur tests. Nevertheless, strength correlated across anatomical sites and, except for the proximal femur, were largely independent of body size effects. In general, males showed greater strength at other sites relative to the metacarpal compared to females, and this sex‐specific difference should be considered in studies comparing male and female subjects.

The greater strength of the radii and femurs relative to the metacarpal for males compared to females may be explained, in part, by the intersection of engineering principles and known sex differences in long bone morphology. As noted above, the strength of short bones like the metacarpal under bending loads depends on the amount of bone (Ct.Ar), whereas the bending strength of radii and femurs, which are long relative to outer width, depend on the distribution of tissue (moment of inertia). Because men have wider long bones and proportionally greater Ct.Ar compared to women even after adjusting for body size^(^ ^24^ ^)^ and because moment of inertia is related to the fourth power of outer bone width but cortical area is related to only the second power of outer width, males and females were expected to show more divergence for moment of inertia (eg, the difference in I_ML_ between males and females is 67% of the average of the two sexes) than for Ct.Ar (eg, the difference in Ct.Ar between males and females is 38% of the average of the two sexes). The dependence of radial and femoral strength on moment of inertia and the dependence of metacarpal strength on Ct.Ar combined with the sex differences in morphology may explain why men showed greater radial and femoral strength relative to metacarpal strength compared to women.

Dimensionless parameters like MCI, ESI, and RCA can be measured in situations when calibration markers are not included in hand radiographs.^(^ ^7^, ^42^, ^43^ ^)^ Our analyses showed strong associations between all dimensionless parameters and strength for females, but not males. We expect much of the sex‐specific differences in the associations between dimensionless parameters and strength can be attributed to the external size dependent out‐of‐plane shape differences (mathematical assumption of circularity). MCI correlated strongly with cortical area and relative cortical area for females, but only relative cortical area for males. Another potential source contributing the sex‐specific differences in morphology–strength associations may be the greater variability of metacarpal strength for females compared to males. The coefficient of variation for second metacarpal strength was 51.1% for females but only 27.6% for males (Table 1). We cannot rule out whether differences in the underlying variation contributed to the stronger associations observed for females. Knowing these limitations may help plan clinical and research studies comparing males and females and screening for fracture risk.^(^ ^43^, ^44^, ^45^ ^)^

Some limitations of this study are important to discuss. First, the cadaveric collection was limited to adult white male and female donors. Additional studies are needed to determine if similar morphology‐strength associations are found for other races/ethnicities with known differences in morphology and whether cortical area can explain racial and ethnic differences in fracture rates.^(^ ^45^ ^)^ Second, multiple anatomical sites were examined, but additional studies are needed to determine how metacarpal strength correlates with other fracture‐prone sites like the distal radius and spine. Third, diaphyseal strength was assessed using standard bending tests, but this loading condition was neither designed nor intended to mimic in situ loads which generally include a combination of bending, torsion, and axial loading modes. The purpose of the mechanical tests conducted herein was to provide general strength measures using methods that are commonly employed by others so our data would be comparable to other studies. Given the short beam structure of the metacarpal and dependence of various loading conditions on similar geometric properties, we would expect similar outcomes if we tested the metacarpals in other loading modes, such as cantilever bending, torsion, or tension. Fourth, the cadaveric tissues examined were limited to donors with no known or observable skeletal disorders. Thus, the data presented herein should be considered representative of healthy, ambulatory individuals. Given that identifying individuals with poor bone health is critical for optimizing treatments and reducing fracture risk,^(^ ^11^ ^)^ additional studies are warranted to determine if the morphology‐strength associations reported herein represent those of populations of clinical interest.^(^ ^44^ ^)^ Finally, we focused exclusively on metacarpal morphology which can be measured from hand radiographs. However, whole‐bone strength also depends on tissue‐level mechanical properties. Thus, future work will need to incorporate tissue‐level mechanical properties into strength estimates, particularly postyield properties which are known to affect strength.^(^ ^15^ ^)^

In conclusion, various parameters used to monitor bone health from hand radiographs were compared to experimentally measured whole‐bone strength at multiple anatomical sites. Cortical area was the best predictor of strength, consistent with engineering principles, and did not show a sex‐specific effect. How changes in metacarpal cortical area relate to changes in bone mass and strength at other anatomical sites has yet to be established. Finding strong associations between changes in metacarpal and hip strength would support broader use of hand radiographs for monitoring fracture risk, particularly in situations when DXA systems are not available.^(^ ^43^, ^46^ ^)^ Assuming a circular morphology was found to generate systematic bias, primarily for males, given that cross‐sectional shape varied significantly with the outer width measured from hand radiographs. Caution is advocated when using dimensionless parameters which showed sex‐specific effects and poor associations between MCI and relative cortical area. The dependence of cross‐sectional shape on the outer bone width measured from a hand radiograph may provide a way to further improve bone health assessments and informed decision making for optimal strength‐building and fracture‐prevention treatment strategies.

## Author Contributions

**Erin M.R. Bigelow:** Conceptualization; data curation; formal analysis; investigation; methodology; project administration; validation; writing – original draft; writing – review and editing. **Robert W. Goulet:** Data curation; investigation; methodology; validation; writing – review and editing. **Antonio Ciarelli:** Investigation; writing – review and editing. **Stephen H Schlecht:** Investigation; writing – review and editing. **David H. Kohn:** Formal analysis; writing – review and editing. **Todd L Bredbenner:** Formal analysis; writing – review and editing. **Sioban D Harlow:** Formal analysis; writing – review and editing. **Carrie A. Karvonen‐Gutierrez:** Formal analysis; writing – review and editing. **Karl J Jepsen:** Conceptualization; formal analysis; funding acquisition; methodology; project administration; supervision; writing – original draft; writing – review and editing.

## Conflicts of Interest

The authors have no conflicts of interest to declare.

## Data availability

All data are available upon request and with approval of an institutional data use agreement.

## Ethics approval

Human tissue use and handling were approved by the University of Michigan Institutional Biosafety Committee and declared exempt by the Institutional Review Board.

## Supporting information


Table S1
Table S2
Table S3
