# Supplementary material for: Sex and External Size Specific Limitations in Assessing Bone Health From Adult Hand Radiographs
Source: JBMR Plus. 2022 Jun 29;6(8):e10653. doi: 10.1002/jbm4.10653 (PMC9382868; doi:10.1002/jbm4.10653)
Supplement: Supplementary file 1 — Table S1 Table S2 Table S3 [file JBM4-6-e10653-s001.docx]

**Supplemental Table 1.** Comparison of summary data for demographic, anthropometric, morphological, and mechanical parameters for men and women (t-test)

|  | **Female** |  | **Male** |  |  |
| --- | --- | --- | --- | --- | --- |
|  | **Mean** | **Stdev** | **Mean** | **Stdev** | **p-value** |
| **Demographic and anthropometric parameters** |  |  |  |  |  |
| Age (years) | 69.4 | 18.9 | 59.9 | 20.8 | 0.066 |
| Height (m) | 1.61 | 0.08 | 1.77 | 0.08 | 0.001 |
| Weight (kg) | 66.60 | 22.40 | 89.20 | 26.58 | 0.001 |
|  |  |  |  |  |  |
| **Hand radiograph parameters** |  |  |  |  |  |
| Paediatric Bone Index, PBI | 5.13 | 1.14 | 6.33 | 0.64 | 0.001 |
| Bone Health Index, BHI | 4.19 | 1.23 | 5.33 | 0.84 | 0.001 |
| Cortical area estimate | 30.99 | 7.77 | 48.46 | 7.62 | 0.001 |
| Summed cortical thickness | 3.19 | 0.95 | 4.38 | 0.66 | 0.001 |
| Metacarpal Index, MCI | 0.40 | 0.12 | 0.48 | 0.09 | 0.015 |
| Exton-Smith Index, ESI | 0.073 | 0.016 | 0.090 | 0.010 | 0.001 |
| Length normalized bone area | 0.007 | 0.002 | 0.009 | 0.002 | 0.001 |
| Relative bone area | 0.67 | 0.16 | 0.65 | 0.10 | 0.465 |
| Length normalized cortical thickness | 0.047 | 0.013 | 0.060 | 0.009 | 0.001 |
|  |  |  |  |  |  |
| **pQCT parameters** |  |  |  |  |  |
| Cortical area, CtAr | 35.27 | 8.07 | 51.20 | 6.79 | 0.001 |
| Moment of inertia, I_ML_ | 178.34 | 38.27 | 329.68 | 101.51 | 0.001 |
|  |  |  |  |  |  |
| **Mechanical Properties** |  |  |  |  |  |
| Second metacarpal strength (Nm) | 5.42 | 2.77 | 9.75 | 2.69 | 0.001 |
| Third metacarpal strength (Nm) | 5.24 | 2.75 | 9.60 | 2.08 | 0.001 |
| Radial diaphysis strength (Nm) | 26.83 | 7.32 | 48.80 | 9.10 | 0.001 |
| Femoral diaphysis strength (Nm) | 200.78 | 73.76 | 346.64 | 80.39 | 0.001 |
| Proximal femur strength (N) | 2814.87 | 1056.12 | 5224.01 | 1419.37 | 0.001 |

Bold values indicate significant correlations or differences in the slope and y-intercept between male and female regressions (ANCOVA).

**Supplemental Table 2.** Linear regression analysis comparing age-adjusted morphological parameters and whole bone strength of the second metacarpal.

| **Parameter** | **Female** | | **Male** | | **ANCOVA** | |
| --- | --- | --- | --- | --- | --- | --- |
|  | **R^2^** | **p-value** | **R^2^** | **p-value** | **slope** | **y-int** |
| **Hand radiograph parameters** |  |  |  |  |  |  |
| Paediatric Bone Index, PBI | **0.498** | **0.001** | **0.369** | **0.001** | 0.375 | **0.002** |
| Bone Health Index, BHI | **0.512** | **0.001** | **0.254** | **0.009** | 0.712 | **0.001** |
| Cortical area estimate | **0.436** | **0.001** | **0.414** | **0.001** | 0.950 | 0.797 |
| Summed cortical thickness | **0.535** | **0.001** | **0.392** | **0.001** | 0.807 | **0.018** |
| Metacarpal Index, MCI | **0.496** | **0.001** | *0.113* | *0.093* | 0.183 | **0.001** |
| Exton-Smith Index, ESI | **0.400** | **0.001** | **0.254** | **0.009** | 0.799 | **0.001** |
| Length normalized bone area | **0.190** | **0.009** | 0.162 | 0.051 | 0.926 | **0.001** |
| Relative bone area | **0.312** | **0.001** | *0.104* | *0.108* | 0.688 | **0.001** |
| Length normalized cortical thickness | **0.448** | **0.001** | **0.249** | **0.009** | 0.811 | **0.001** |
| **pQCT parameters** |  |  |  |  |  |  |
| Cortical area, CtAr | **0.697** | **0.001** | **0.592** | **0.001** | 0.650 | 0.508 |
| Moment of inertia, I_ML_ | **0.497** | **0.001** | **0.243** | **0.011** | **0.006** | n/a |

Bold values indicate significant correlations or differences in the slope and y-intercept between male and female regressions (ANCOVA). Italic font indicates a change in significance compared to the unadjusted data.

**Supplemental Table 3.** Linear regression analysis comparing various age-adjusted morphological parameters to whole bone strength across multiple sites for A) female and B) male cadaveric bones

**Supplemental 3A. Female**

| **Parameter** | **MC3** | | **Radius** | | **Femur** | | **Proximal Femur** | |
| --- | --- | --- | --- | --- | --- | --- | --- | --- |
|  | **R^2^** | **p-value** | **R^2^** | **p-value** | **R^2^** | **p-value** | **R^2^** | **p-value** |
| **Hand radiographs** |  |  |  |  |  |  |  |  |
| Paediatric Bone Index, PBI | **0.439** | **0.001** | **0.491** | **0.001** | **0.402** | **0.001** | **0.231** | **0.005** |
| Bone Health Index, BHI | **0.472** | **0.001** | **0.420** | **0.001** | **0.397** | **0.001** | **0.229** | **0.005** |
| Cortical area estimate | **0.302** | **0.001** | **0.393** | **0.001** | **0.329** | **0.001** | **0.269** | **0.002** |
| Summed cortical thickness | **0.465** | **0.001** | **0.420** | **0.001** | **0.402** | **0.001** | **0.257** | **0.003** |
| Metacarpal Index, MCI | **0.524** | **0.001** | **0.385** | **0.001** | **0.377** | **0.001** | **0.189** | **0.011** |
| Exton-Smith Index, ESI | **0.321** | **0.001** | **0.448** | **0.001** | **0.340** | **0.001** | **0.198** | **0.009** |
| Length normalized bone area | **0.138** | **0.001** | **0.253** | **0.002** | **0.165** | **0.021** | **0.133** | **0.037** |
| Relative bone area | **0.451** | **0.001** | **0.266** | **0.002** | **0.175** | **0.017** | **0.127** | **0.042** |
| Length normalized cortical thickness | **0.382** | **0.001** | **0.408** | **0.001** | **0.362** | **0.001** | **0.211** | **0.007** |
| **pQCT** |  |  |  |  |  |  |  |  |
| Cortical area, CtAr | **0.631** | **0.001** | **0.591** | **0.001** | **0.321** | **0.001** | **0.250** | **0.003** |
| Moment of inertia, I_ML_ | **0.278** | **0.001** | **0.438** | **0.001** | *0.105* | *0.071* | **0.120** | **0.049** |

MC3 = third metacarpal. Bold values indicate significant correlations. Italic font indicates a change in significance compared to the unadjusted data.

**Supplemental 3B. Male**

| **Parameter** | **MC3** | | **Radius** | | **Femur** | | **Proximal Femur** | |
| --- | --- | --- | --- | --- | --- | --- | --- | --- |
|  | **R^2^** | **p-value** | **R^2^** | **p-value** | **R^2^** | **p-value** | **R^2^** | **p-value** |
| **Hand radiographs** |  |  |  |  |  |  |  |  |
| Paediatric Bone Index, PBI | **0.418** | **0.001** | 0.018 | 0.509 | **0.180** | **0.035** | 0.067 | 0.222 |
| Bone Health Index, BHI | **0.312** | **0.003** | 0.001 | 0.998 | 0.079 | 0.174 | 0.066 | 0.225 |
| Cortical area estimate | **0.443** | **0.001** | **0.211** | **0.018** | **0.352** | **0.002** | 0.025 | 0.463 |
| Summed cortical thickness | **0.459** | **0.001** | 0.020 | 0.489 | **0.166** | **0.044** | 0.073 | 0.202 |
| Metacarpal Index, MCI | *0.149* | *0.052* | 0.032 | 0.383 | 0.004 | 0.756 | 0.057 | 0.263 |
| Exton-Smith Index, ESI | **0.307** | **0.003** | 0.026 | 0.433 | **0.199** | **0.025** | 0.038 | 0.363 |
| Length normalized bone area | **0.224** | **0.020** | 0.088 | 0.160 | **0.263** | **0.010** | 0.006 | 0.726 |
| Relative bone area | *0.148* | *0.052* | 0.017 | 0.529 | 0.001 | 0.832 | 0.042 | 0.336 |
| Length normalized cortical thickness | **0.303** | **0.004** | 0.004 | 0.771 | 0.122 | 0.087 | 0.053 | 0.277 |
| **pQCT** |  |  |  |  |  |  |  |  |
| Cortical area, CtAr | **0.413** | **0.001** | *0.106* | *0.105* | **0.290** | **0.006** | 0.032 | 0.404 |
| Moment of inertia, I_ML_ | **0.178** | **0.032** | **0.207** | **0.019** | **0.161** | **0.047** | 0.001 | 0.874 |

MC3 = third metacarpal. Bold values indicate significant correlations. Italic font indicates a change in significance compared to the unadjusted data.
